# Glomerular crescents are associated with the risk of type 2 diabetic kidney disease progression: a retrospective cohort study

**DOI:** 10.1186/s12882-024-03578-y

**Published:** 2024-05-20

**Authors:** Sohyun Bae, Donghwan Yun, Sung Woo Lee, Jong Hyun Jhee, Jung Pyo Lee, Tae Ik Chang, Jieun Oh, Young Joo Kwon, Sung Gyun Kim, Hajeong Lee, Dong Ki Kim, Kwon Wook Joo, Kyung Chul Moon, Ho Jun Chin, Seung Seok Han

**Affiliations:** 1 Department of Internal Medicine, Seoul National University College of Medicine, 103 Daehak-Ro, Jongno-Gu, Seoul, 03080 Korea; 2 Department of Biomedical Sciences, Seoul National University College of Medicine, Seoul, Korea; 3 Department of Internal Medicine, Uijeongbu Eulji Medical Center, Gyeonggi-Do, Korea; 4 Department of Internal Medicine, Gangnam Severance Hospital, Seoul, Korea; 5 Division of Nephrology, Seoul National University Boramae Medical Center, Seoul, Korea; 6 Department of Internal Medicine, National Health Insurance Service Medical Center Ilsan Hospital, Gyeonggi-Do, Korea; 7 Department of Internal Medicine, Hallym University Kangdong Sacred Heart Hospital, Seoul, Korea; 8 Department of Internal Medicine, Korea University Medical Center, Seoul, Korea; 9 Department of Internal Medicine, Hallym University Sacred Heart Hospital, Gyeonggi-Do, Korea; 10 Department of Pathology, Seoul National University College of Medicine, Seoul, Korea; 11 Department of Internal Medicine, Seoul National University Bundang Hospital, Seoul National University College of Medicine, 82 Gumi-Ro, 173-Beon-Gil, Bundang-Gu, Seongnam-Si, Gyeonggi-Do, 03080 Korea

**Keywords:** Biopsy, Crescent, Diabetic kidney disease, Diabetic nephropathy, Histology

## Abstract

**Background:**

Diabetic kidney disease (DKD) stands as the predominant cause of chronic kidney disease and end-stage kidney disease. Its diverse range of manifestations complicates the treatment approach for patients. Although kidney biopsy is considered the gold standard for diagnosis, it lacks precision in predicting the progression of kidney dysfunction. Herein, we addressed whether the presence of glomerular crescents is linked to the outcomes in patients with biopsy-confirmed type 2 DKD.

**Methods:**

We performed a retrospective evaluation, involving 327 patients diagnosed with biopsy-confirmed DKD in the context of type 2 diabetes, excluding cases with other glomerular diseases, from nine tertiary hospitals. Hazard ratios (HRs) were calculated using a Cox regression model to assess the risk of kidney disease progression, defined as either ≥ 50% decrease in estimated glomerular filtration rates or the development of end-stage kidney disease, based on the presence of glomerular crescents.

**Results:**

Out of the 327 patients selected, ten patients had glomerular crescents observed in their biopsied tissues. Over the follow-up period (median of 19 months, with a maximum of 18 years), the crescent group exhibited a higher risk of kidney disease progression than the no crescent group, with an adjusted HR of 2.82 (1.32–6.06) (*P* = 0.008). The presence of heavy proteinuria was associated with an increased risk of developing glomerular crescents.

**Conclusion:**

The presence of glomerular crescents is indeed linked to the progression of type 2 DKD. Therefore, it is important to determine whether there is an additional immune-mediated glomerulonephritis requiring immunomodulation, and it may be prudent to monitor the histology and repeat a biopsy.

**Supplementary Information:**

The online version contains supplementary material available at 10.1186/s12882-024-03578-y.

## Background

Diabetic kidney disease (DKD) has become the main cause of chronic kidney disease (CKD) and end-stage kidney disease (ESKD) because of the significant proportion of people with diabetes worldwide [1]. With diabetes affecting 10.5% of individuals aged 20 to 79 years (approximately over 530 million people) worldwide, its association with an approximately twofold risk of CKD compared to patients without diabetes highlights the importance of addressing DKD [2, 3]. Nonetheless, given the multitude of factors influencing the progression of DKD, the current diagnostic and treatment options remain somewhat limited. This underscores the importance of exploring novel mechanisms and developing more effective approaches for both diagnosis and treatment [4].

The diagnosis of DKD is primarily based on clinical features. In certain cases, a kidney biopsy may be required to confirm the diagnosis, because there is a possibility of kidney disease other than DKD [5–7]. The Renal Pathology Society (RPS) classification, introduced in 2010, provides a histological classification of DKD [8]. This scoring system incorporates histological findings, including glomerular, tubulointerstitial, and vascular lesions. Several studies have shown that specific histological characteristics of DKD are associated with a worse prognosis, while other studies have found no significant association with kidney prognosis [4, 9, 10]. In a study estimating the 5-year renal survival rate of enrolled patients, higher RPS classification scores were associated with a worse outcome [9]. Interstitial fibrosis and tubular atrophy, indicating chronic and irreversible injury, are strong predictors of kidney dysfunction and poor prognosis of DKD [10, 11]. However, in some studies, aspects of glomerular lesions, such as mesangial expansion or nodular sclerosis, were not significantly associated with diabetic patients with overt proteinuria [11]. Another retrospective cohort study revealed that the type of diabetic glomerulosclerosis, whether diffuse or nodular, did not predict the progression of kidney disease in diabetic patients [12].

Crescents, which serve as a histological parameter of severe glomerular injury involving immune cells, fibrin, and complements, are not included in the histological classification of DKD, because these are very rare findings [13, 14]. The presence of glomerular crescents is a characteristic feature of rapidly progressive glomerulonephritis and can be observed in the severe forms of various types of glomerulonephritis, including postinfectious glomerulonephritis, membranoproliferative glomerulonephritis, lupus nephritis, and immunoglobulin A nephropathy [14–16]. While glomerular crescents have primarily been identified in the acute form of glomerulonephritis, there have been reports of their occurrence in isolated cases of DKD [17–21]. Some single-center cohort studies have examined the relationship between glomerular crescents and a poorer kidney prognosis in patients with type 2 DKD [22–25]. However, there has been no multicenter cohort study investigating the association between glomerular crescents and the clinical prognosis in type 2 DKD patients. Herein, the present study aimed to assess the clinical implication of glomerular crescents in biopsy-confirmed DKD among type 2 diabetic patients, along with its risk factors, using data from a multicenter cohort conducted in South Korea. Furthermore, we sought to enhance our understanding of this relationship by pooling previously published data.

## Methods

### Patient and data collection

**Fig. 1:**
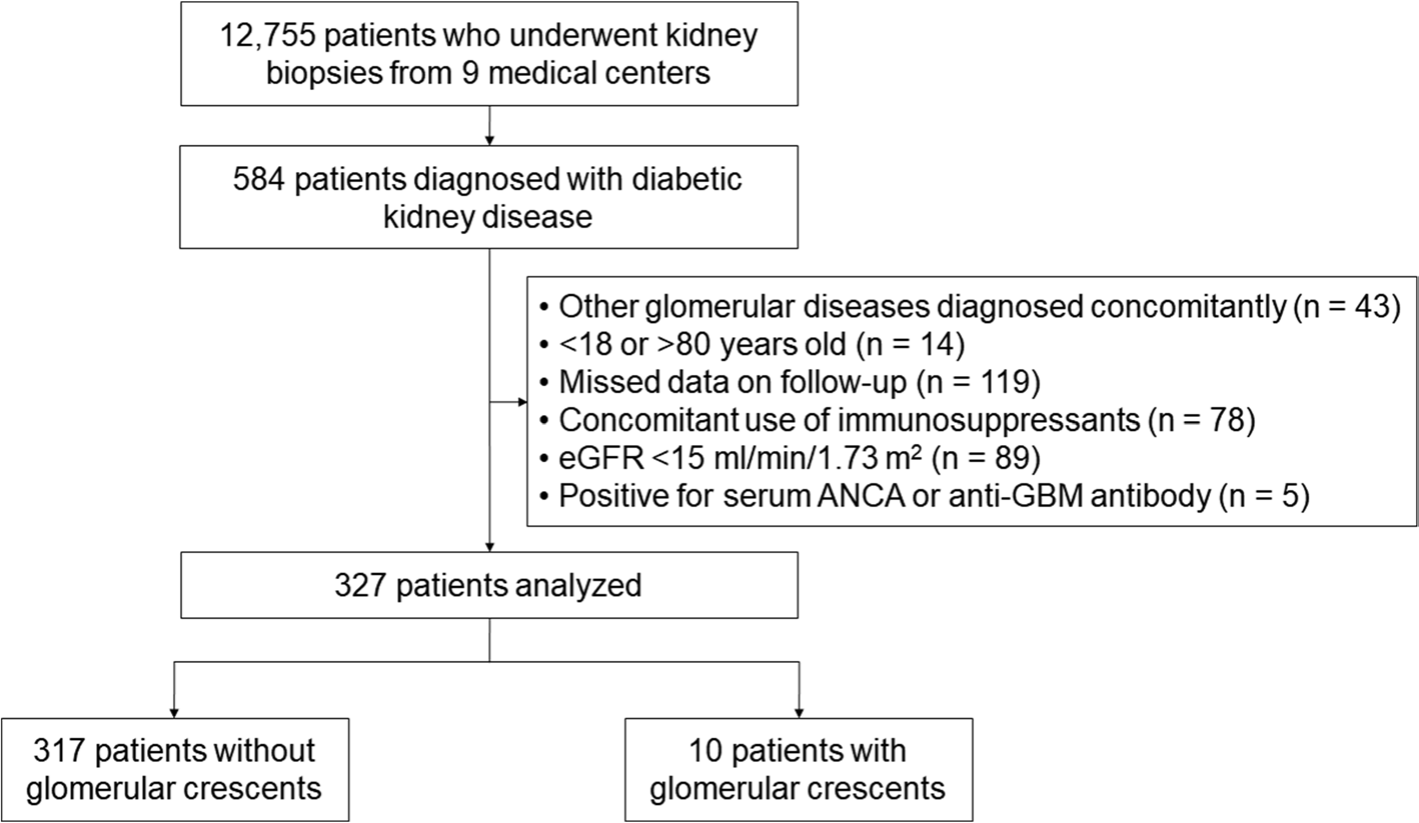
Flowchart for patient inclusion and exclusion. eGFR, estimated glomerular filtration rate; ANCA, anti-neutrophil cytoplasmic antibody; GBM, glomerular basement membrane

A total of 12,755 patients who underwent kidney biopsy between 1979 and 2018 were retrospectively reviewed from 9 tertiary hospitals in South Korea. Among them, patients who had kidney disease other than DKD were excluded (*n* = 12,171). Additionally, patients who had concomitant other glomerular disease (*n* = 43), who were under the age of 18 years or over the age of 80 years at the time of biopsy (*n* = 14), who used immunosuppressive agents after biopsy *(n* = 78), who had an estimated glomerular filtration rate (eGFR) of < 15 ml/min/1.73 m^2^ (*n* = 89), who were positive for serum anti-neutrophil cytoplasmic or anti-glomerular basement membrane antibodies (*n* = 5), and whose laboratory data were incomplete (*n* = 119) were excluded. Finally, a total of 327 patients were included in the analysis (Fig. 1). The study protocol was approved by the institutional review board of the Seoul National University Hospital (no. H-2209–012-1355) and was conducted in accordance with the ethical standards outlined in the Declaration of Helsinki. Written consent was waived of the study by the institutional review board of the Seoul National University Hospital.

Clinical data at the time of biopsy, such as age, sex, BMI, and comorbidities (e.g., hypertension, cardiovascular disease, and malignancy) were obtained. Blood laboratory data, such as hemoglobin, albumin, total cholesterol, blood urea nitrogen, and creatinine levels, were obtained. The estimated glomerular filtration rate (eGFR) was calculated using the Chronic Kidney Disease Epidemiology Collaboration Equation [26]. Proteinuria was evaluated using a random urine protein-to-creatinine ratio. The values of eGFR were traced until either the patient was lost to follow-up or reached the end date of the study (February 2019).

The pathological data for this study were sourced from pathology reports, as provided by pathologists at each participating hospital. The biopsied tissues were evaluated for three compartments: glomeruli, tubulointerstitium, and vessels. Glomerular lesions included global glomerulosclerosis and mesangial expansion. Regarding the tubulointerstitial compartment, the severity of interstitial fibrosis, interstitial inflammation, and tubular atrophy were assessed and graded with light microscopy findings. They were evaluated by each pathologist at the centers based on the RPS classification as a categorical variable, graded as none (0%), mild (> 0% to < 25%), moderate (25% to 50%), or severe (> 50%) [8]. Vessel lesions were assessed by the presence of arteriosclerosis. Global glomerulosclerosis was expressed as a percentage among all glomeruli, while the grades for mesangial matrix, interstitial fibrosis, interstitial inflammation, and tubular atrophy were classified as none, mild, moderate, and severe. Regarding the severity of pathological findings, we classified the severity of findings in each specimen by reviewing the reports evaluated and written by pathologists based on the RPS classification. Arteriosclerosis was indicated as either present or absent. Glomerular crescents were characterized by the presence of multiple layers of proliferating cells within Bowman’s space. The presence of staining for immunoglobulins, complements, fibrinogen, and light chains was detected and graded. The degree of immunofluorescence was graded on a scale of 0–4 by renal pathologists at each center.

Kidney disease progression as an outcome was defined as a decrease of > 50% in eGFR from baseline or the development of ESKD. Follow-up was censored when patients reached the end date of the study or at the last available measurement.

#### Statistical analysis

Patient information was subjected to descriptive statistical analyses, and the data are expressed as the mean ± standard deviation when exhibiting a normal distribution and as medians with interquartile ranges when lacking a normal distribution for continuous variables. The Kolmogorov–Smirnov test was employed to analyze the normality of distribution. For categorical variables, the data are expressed as a number (%). Categorical variables were compared using the chi-square test or Fisher’s exact test, while continuous variables were compared using Student’s t test or the Mann–Whitney U test according to their distributions.

The association between the presence of glomerular crescents and kidney disease progression was evaluated using Kaplan–Meier survival curves with a log-rank test. Univariate and multivariate Cox proportional hazard analyses were performed to assess the effects of various variables on kidney disease progression. Additionally, multivariate Cox proportional hazard analyses were conducted to determine hazard ratios (HRs) and confidence intervals (CIs) for the outcome.

If one or more glomerular crescents were observed in the biopsied tissue, the sample was classified into the crescent group. To create Kaplan–Meier survival curves and perform Cox proportional hazard analyses, patients were divided into two groups based on the presence of glomerular crescents. Stepwise Cox proportional hazard analyses were conducted using four different models. Model 1 was unadjusted, while Model 2 was adjusted for age and sex. Variables with *P* < 0.1 in Table 1 (specifically, serum hemoglobin, serum albumin, and random urine protein-to-creatinine ratio) were incorporated into the multivariate Cox proportional Model 3, and Model 4 included adjustments for all variables. To pinpoint risk factors for glomerular crescents, logistic regression with backward stepwise selection was used. All statistical analyses were performed using the R program (version 4.2.3) and a *P* value less than 0.05 was considered to indicate a statistically significant difference.

**Table 1:** Baseline characteristics of patients

Variables	Total (*n* = 327)	Crescent (*n* = 10)	No crescent (*n* = 317)	*P*
Age (years)	51.2 ± 12.1	49.6 ± 10.3	51.2 ± 12.2	0.672
Male (%)	209 (63.9)	7 (70.0)	202 (63.7)	0.942
Body mass index (kg/m^2^)	24.3 (22.1–26.9)	26.9 (22.1–27.0)	24.3 (22.1–26.6)	0.448
Comorbidities (%)				
Hypertension	275 (84.1)	8 (80.0)	267 (84.2)	0.999
Cardiovascular disease	32 (9.8)	1 (10.0)	31 (9.8)	0.999
Malignancy	30 (9.2)	1 (10.0)	29 (9.1)	0.999
Blood findings				
Hemoglobin (g/dl)	11.1 ± 2.1	9.7 ± 1.0	11.2 ± 2.1	0.001
Albumin (g/dl)	3.1 ± 0.7	2.7 ± 0.7	3.2 ± 0.7	0.081
Total cholesterol (mg/dl)	188 (151–233)	184 (117–214)	188 (151–233)	0.485
Blood urea nitrogen (mg/dl)	23.0 (17.0–31.0)	25.5 (17.0–37.0)	23.0 (17.0–31.0)	0.672
Creatinine (mg/dl)	1.4 (1.0–2.0)	1.9 (1.4–2.4)	1.4 (1.0–2.0)	0.128
eGFR (ml/min/1.73 m^2^)	51.1 (34.2–72.0)	41.8 (24.2–55.2)	51.1 (34.4–73.5)	0.153
uPCR (g/g)	4.1 (1.9–7.8)	10.5 (7.1–12.8)	4.0 (1.9–7.7)	0.043

Data are presented as mean ± standard deviation or median (interquartile rage) for continuous variables and the number (%) for categorical variables

*eGFR* estimated glomerular filtration rate, *uPCR* random urine protein-to-creatinine ratio

#### Study selection, data extraction, and statistical analysis for meta-analysis

A meta-analysis was performed with previously published data in addition to ours. To identify relevant studies, we searched databases, such as PubMed, Excerpta Medica Database, Cochrane library, and Web of Science, on September 2023 with medical subject heading terms and text words in multiple combinations: “diabetic nephropathy”, “diabetic kidney disease”, “crescent”, and “extracapillary hypercellularity.” Accordingly, 4 retrospective cohort studies including DKD patients were used because information on HRs of the ESKD outcome and the presence of glomerular crescents were available [22–25]. The pooled effect estimates and associated 95% CIs were calculated using a common effect model. This statistical analysis was also performed using the R program (version 4.2.3).

## Results

### Patient characteristics

The median age of the patients was 51.2 ± 12.1 years old, and 63.9% of patients were male. Out of the total, ten patients (3.1%) displayed glomerular crescents in their biopsied tissues. The median percentage of glomerular crescents among all glomeruli was 9.9% (interquartile range, 4.3%–12.3%; maximum, 36%). Among the ten patient specimens analyzed, the distribution of crescent types was as follows: 30% cellular, 20% fibrocellular, and 30% fibrous. Additionally, there was one instance of combined types and another where the type was unspecified. In comparison to patients without crescents, those with crescents had higher levels of proteinuria and lower hemoglobin levels. Other clinical variables were not different between the two groups. Table 1 provides the comparison of baseline clinical and laboratory findings between these groups.

Comparisons of light microscopy and immunofluorescence findings between the crescent and no crescent groups are presented in Tables 2 and 3, respectively. No significant disparities were observed between the two groups regarding the percentages of global glomerulosclerosis, the grade of mesangial expansion, interstitial fibrosis, tubular atrophy, interstitial inflammation, or the presence of arteriosclerosis. In the crescent group, Kimmelstiel-Wilson nodules, indicative of nodular glomerulosclerosis, were identified in 6 out of 10 specimens (60%), while in the control cases, nodules were identified in 120 out of 317 specimens (37.9%). Among the 6 specimens where nodules were identified, 2 were classified as cellular, 1 as fibrous, another as fibrous, 1 as combined type, and 1 as unspecified. Furthermore, it is noted that mesangial expansion and nodular diabetic glomerulosclerosis are delineated as separate entities in the pathology reports. In the assessment of immunofluorescence findings, significant differences were noted in the intensity of fibrinogen deposition between the two groups.

**Table 2:** Light microscopy findings according to the presence of glomerular crescents

Variables	Total (*n* = 327)	Crescent (*n* = 10)	No crescent (*n* = 317)	*P*
Global glomerulosclerosis				0.194
< 20%	117 (35.8)	2 (20.0)	115 (36.3)	
20%–49%	138 (42.2)	7 (70.0)	131 (41.3)	
≥ 50%	72 (22.0)	1 (10.0)	71 (22.4)	
Mesangial expansion				0.798
None	102 (31.2)	3 (30.0)	99 (31.2)	
Mild	102 (31.2)	2 (20.0)	100 (31.5)	
Moderate	40 (12.2)	2 (20.0)	38 (12.0)	
Severe	83 (25.4)	3 (30.0)	80 (25.2)	
Interstitial fibrosis				0.356
None	19 (5.8)	1 (10.0)	18 (5.7)	
Mild	117 (35.8)	1 (10.0)	116 (36.6)	
Moderate	107 (32.7)	5 (50.0)	102 (32.2)	
Severe	84 (25.7)	3 (30.0)	81 (25.6)	
Tubular atrophy				0.189
None	12 (3.7)	0 (0.0)	12 (3.8)	
Mild	103 (31.5)	1 (10.0)	102 (32.2)	
Moderate	111 (33.9)	3 (30.0)	108 (34.1)	
Severe	101 (30.9)	6 (60.0)	95 (30.0)	
Interstitial inflammation				0.579
None	45 (13.8)	45 (14.2)	0 (0.0)	
Mild	148 (45.3)	5 (50.0)	143 (45.1)	
Moderate	94 (28.7)	4 (40.0)	90 (28.4)	
Severe	40 (12.2)	1 (10.0)	39 (12.3)	
Arteriosclerosis	191 (58.4)	5 (50.0)	186 (58.7)	0.824

Data are presented as the number (%)

**Table 3:** Immunofluorescence findings according to the presence of glomerular crescents

Variables	Total (*n* = 327)	Crescent (*n* = 10)	No crescent (*n* = 317)	*P*
C3 deposition				0.655
None or trace	292 (89.3)	8 (80.0)	284 (89.6)	
≥ 1 +	35 (10.7)	2 (20.0)	33 (10.4)	
C1q deposition				0.999
None or trace	316 (96.6)	10 (100.0)	306 (96.5)	
≥ 1 +	11 (3.4)	0 (0.0)	11 (3.5)	
C4d deposition				0.999
None or trace	324 (99.1)	10 (100.0)	314 (99.1)	
≥ 1 +	3 (0.9)	0 (0.0)	3 (0.9)	
Fibrinogen deposition				0.016
None or trace	318 (97.2)	8 (80.0)	310 (97.8)	
≥ 1 +	9 (2.8)	2 (20.0)	7 (2.2)	
IgA deposition				0.999
None or trace	305 (93.3)	9 (90.0)	296 (93.4)	
≥ 1 +	22 (6.7)	1 (10.0)	21 (6.6)	
IgG deposition				0.165
None or trace	290 (88.7)	7 (70.0)	283 (89.3)	
≥ 1 +	37 (11.3)	3 (30.0)	34 (10.7)	
IgM deposition				0.243
None or trace	285 (87.2)	7 (70.0)	278 (87.7)	
≥ 1 +	42 (12.8)	3 (30.0)	39 (12.3)	
κ light chain deposition				0.999
None or trace	310 (94.8)	9 (90.0)	301 (95.0)	
≥ 1 +	17 (5.2)	1 (10.0)	16 (5.0)	
λ light chain deposition				0.999
None or trace	307 (93.9)	9 (90.0)	298 (94.0)	
≥ 1 +	20 (6.1)	1 (10.0)	19 (6.0)	

Data are presented as the number (%)

*C3* complement 3, *C1q* complement 1q, *C4d* complement 4d, *IgA* immunoglobulin A, *IgG* immunoglobulin G, *IgM* immunoglobulin M

### Kidney outcome according to the presence of glomerular crescents

**Fig. 2:**
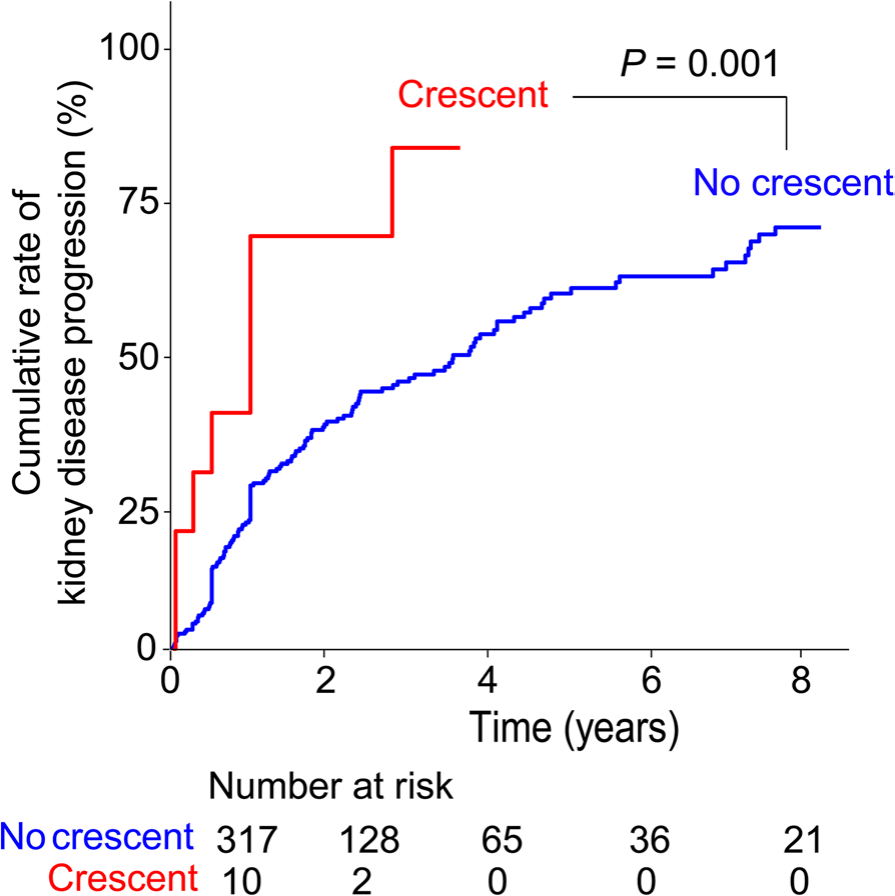
Kaplan–Meier curve showing kidney disease progression rates according to the presence of glomerular crescents

The median duration of follow-up was 19 months (interquartile range, 6–42 months; maximum, 18 years). Over this period, kidney disease progression was identified in 162 (49.5%) patients. Supplementary Table 1 presents the clinical and histological parameters that influenced kidney disease progression, apart from the presence of glomerular crescents. Factors associated with the progression of kidney disease included young age, hypertension, hypoalbuminemia, azotemia, proteinuria, global glomerulosclerosis, and complement 3 deposition. Out of the 327 patients, 10 (3.1%) had glomerular crescents in their biopsied tissues. Kaplan–Meier survival analysis showed that the crescent group exhibited a worse kidney outcome than the no crescent group (*P* = 0.001) (Fig. 2). In the univariate Cox proportional hazard analysis, the crescent group had a higher risk of kidney disease progression than the no crescent group, with an unadjusted HR of 2.82 (1.315–6.061) (*P* = 0.008) (Table 4). In multivariate analysis models, the crescent group consistently exhibited a higher risk of kidney disease progression than the no crescent group.

**Table 4:** Hazard ratio of kidney outcome in patents with crescents compared with those without crescents

	Hazard ratio (95% confidence interval)	*P*
Model 1	2.82 (1.315–6.061)	0.008
Model 2	2.79 (1.292–6.005)	0.009
Model 3	2.37 (1.021–5.485)	0.045
Model 4	4.81 (1.498–15.449)	0.008

Model 1: Unadjusted

Model 2: Adjusted for age and sex

Model 3: Adjusted for variables with *P* < 0.1 in Table 1, such as serum hemoglobin, serum albumin, and random urine protein-to-creatinine ratio

Model 4: Adjusted for all variables

### Factors related to glomerular crescents

Upon applying a multivariable logistic regression model with backward stepwise selection, heavy proteinuria was associated with the presence of glomerular crescents (Table 5). Other clinical and blood laboratory data did not appear to be related to glomerular crescents.

**Table 5:** Risk factors related to glomerular crescents

Variables	Unadjusted OR (95% CI)	*P*	Adjusted OR (95% CI)^a^	*P*
Age (per 1 year)	0.99 (0.939–1.042)	0.672		
Male (vs. female)	0.75 (0.191–2.968)	0.684		
Body mass index (per 1 kg/m^2^)	1.08 (0.907–1.288)	0.385		
Hypertension (vs. none)	0.75 (0.154–3.632)	0.720		
Cardiovascular disease (vs. none)	1.03 (0.126–8.362)	0.982		
Malignancy (vs. none)	1.10 (0.135–9.020)	0.927		
Hemoglobin (per 1 g/dl)	0.67 (0.466–0.974)	0.036		
Albumin (per 1 g/dl)	0.47 (0.198–1.112)	0.086		
Cholesterol (per 1 mg/dl)	1.00 (0.987–1.007)	0.561		
Blood urea nitrogen (per 1 mg/dl)	1.01 (0.958–1.058)	0.795		
Creatinine (per 1 mg/dl)	1.60 (0.836–3.059)	0.156		
uPCR (per 1 g/g)	1.13 (1.025–1.245)	0.014	1.19 (1.034–1.358)	0.014

*OR* odds ratio, *CI* confidence interval, *uPCR* random urine protein-to-creatinine ratio

^a^Adjusted for all variables with backward stepwise selection

### Pooled analysis with previous data

**Fig. 3:**
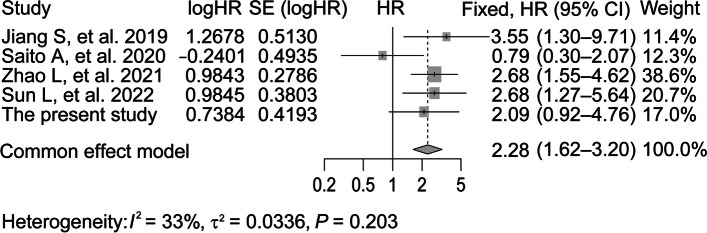
Forest plot showing the meta-analyzed estimate of the HR for ESKD associated with glomerular crescents. SE, standard error; CI, confidence interval

Recognizing that the proportion of patients in the crescent group was relatively low, at 3.1%, we conducted a pooled analysis by combining data from other relevant literature to enhance the clinical significance of glomerular crescents in DKD as comprehensively as possible. Retrospective cohort studies that involved crescent formation in type 2 DKD patients and provided HR values for crescents from multivariate Cox proportional hazard analyses were selected. Ultimately, four studies were included and the extracted data included first author, year of publication, country, sample size, HR value of crescents from multivariate Cox proportional hazard models, and the corresponding 95% confidence interval (Supplementary Table 2). Through a meta-analysis that combined 4 previous studies on DKD and the present study (*n* = 984), glomerular crescents were determined to be a risk factor for progression to ESKD, with an HR of 2.28 (1.62–3.20) (*I*^2^ = 33%; *P* for heterogeneity = 0.203) (Fig. 3).

## Discussion

Glomerular crescents, a rare finding in DKD, are indicative of severe glomerular injury. In a multicenter cohort study involving type 2 diabetic patients with biopsy-proven DKD, glomerular crescents were found to be associated with kidney disease progression. This association persisted even after adjusting for multiple clinical and histological factors. Heavy proteinuria was identified as a risk factor for the development of glomerular crescents. Furthermore, according to a meta-analysis, patients with crescents were at a higher risk of progression to ESKD than those without crescents.

A glomerular crescent is a common finding in patients with rapidly progressive glomerulonephritis and serves as a prognostic factor for acute or subacute kidney injuries [27]. It is characterized by the accumulation of cellular or fibrotic materials in Bowman’s space [28]. Glomerular crescents form as a result of injury to the glomerular basement membrane, which triggers complement activation, inflammation, and the leakage of plasma proteins such as fibrinogen into Bowman's space [29]. This process stimulates the proliferation of parietal epithelial cells and the infiltration of inflammatory cells, culminating in the formation of a crescent-shaped structure that compresses the glomerulus and compromises kidney function [30, 31]. The International Society of Nephrology/Renal Pathology Society classification for lupus nephritis proposes categorizing crescents as cellular, fibrocellular, or fibrous based on the proportions of cells, fibrin, and fibrous matrix [16]. Research indicates that cellular crescents arise from parietal epithelial cells on Bowman’s capsule and contribute to a decline in the glomerular filtration rate, but certain cases suggest that cellular crescents may be reversible with appropriate treatment [32]. The proliferation of parietal epithelial cells can lead to a mesenchymal transition to fibrous crescents that results, resulting in the release of extracellular matrix. These changes are irreversible, indicating that targeting interstitial fibrosis therapeutically does not restore lost nephron function [32].

Meanwhile, the complement cascade is known to be involved in the pathophysiology of DKD [33–35]. Exudative lesions or hyaline caps can be identified in the kidney of DKD similar to other kidney diseases [36]. In addition to the rupture of the glomerular basement membrane and the subsequent complement cascade mentioned earlier, by establishing a physical connection between the glomerular tuft and Bowman's capsule through the spreading of podocytes, these bridges may initiate the proliferation of parietal epithelial cells, thereby contributing to crescent formation [37]. Interestingly, unlike the crescents observed in chronic glomerulonephritis, which are associated with rupture of the glomerular basement membrane, some studies suggest that crescents in diabetic glomerulosclerosis are composed of a mix of parietal epithelial cells and podocytes. This implies the potential transdifferentiation of these cells in response to glomerular injury, occurring without glomerular basement membrane rupture [18, 21, 37, 38].

A single Chinese cohort with DKD cases showed a positive correlation between crescents and complement 3 deposition as well as disease progression, suggesting that an abnormal complement system may be involved in the formation of crescents [22]. In the present study, we observed significant differences in fibrinogen deposition intensities between the crescent and no-crescent groups. These findings support the concept that fibrinogen plays a significant role in the pathogenesis of crescentic glomerulonephritis [39]. Diabetic nodules may increase the risk of capillary microaneurysms, leading to the formation of crescents [40]. Further preclinical and clinical studies investigating the mechanism and factors underlying crescent formation in DKD are warranted.

Kidney biopsy can hold significant importance in diagnosing DKD, providing a definitive confirmation of the diagnosis and aiding in the differentiation of DKD from other kidney diseases, especially in patients exhibiting atypical clinical features [41]. Additionally, it can also offer crucial histological information about the severity and extent of kidney damage, which can guide personalized treatment plans and prognostic assessments, aiding clinicians in monitoring disease progression. The present study results may support above issue, although careful consideration of the associated risks of biopsy should be undertaken.

Several factors, such as older age, high blood pressure, and azotemia, serve as risk factors for the progression of DKD [4, 42]. Some histological features, such as interstitial fibrosis, tubular atrophy, and interstitial inflammation in diabetic kidneys, might be associated with a poor renal prognosis [4, 9, 11, 25]. We sought to elucidate the effects of crescents on kidney disease progression after correcting of the above clinical and histological parameters. Furthermore, we analyzed risk factors associated with the presence of glomerular crescents, which was heavy proteinuria. Thus, it may be recommended that physicians monitor patients exhibiting such feature for the potential occurrence of glomerular crescents and aggravating kidney dysfunction.

Although this study provides insightful information, it presents certain limitations. One of the principal limitations of this study is the small number of patients with glomerular crescents. To address this limitation and enhance the robustness of our findings, we conducted a pooling analysis. This approach was aimed at consolidating data across multiple studies, thereby attempting to mitigate the impact of the limited sample size on our ability to draw meaningful conclusions. Because this study was retrospective in nature, causality with kidney disease progression could not be determined. Unmeasured biases and confounding factors could have interfered with correlation analyses. Continuous fluctuations in biochemical factors and alterations in practice could be related to kidney disease progression but were not considered in the study. The influence of acute kidney injury on follow-up was not known, which could have affected the results. While an ideal approach would involve a uniform review of all tissue samples by a single pathologist to ensure accuracy and consistency in the pathological assessment, the retrospective nature of our study precluded this. Because this study was retrospective and depended solely on pathology texts, obtaining tissue slides poses a challenge. This limitation hampers further investigation in areas such as examining the presence of urinary space collagen with fibrotic crescents to gauge diabetes-related injury or determining the location of fibrinogen deposition in immunofluorescence and other changes (e.g., misdirected filtration and fibrinoid exudation) to speculate on the causes of crescent formation [43–45]. It is pertinent to note that all biopsies underwent review by pathologists at each center, indicating the reliability of histological reports. In addition, while we cannot entirely rule out the presence of ANCA-negative crescentic glomerulonephritis in the crescent group, the predominant histological evidence strongly indicates a definitive diagnosis of DKD. This finding is crucial for understanding the relationship between the presence of glomerular crescents in DKD and kidney outcome. Nevertheless, diligent efforts to rule out the possibility of crescentic glomerulonephritis and to initiate timely treatment when such a possibility cannot be completely excluded are essential.

The present study did not determine the pathophysiology of glomerular crescents, which could further discern the association between crescents and kidney disease progression. There was a lack of data on the use of treatment agents, such as renin-angiotensin system inhibitors and sodium-glucose cotransporter 2 inhibitors, which are frequently used in patients with type 2 DKD, as well as other important clinical prognostic factors such as the duration of diabetes mellitus, microscopic hematuria and hemoglobin A1c. It is challenging to establish unified guidelines for kidney biopsy in patients with a DKD diagnosis in this multicenter retrospective cohort study. It would be worthwhile to conduct other studies that adjust for the above issues in the future.

In contrast to other forms of glomerulonephritis, the precise impact of crescents on the prognosis of type 2 DKD has not been fully established. We endeavored to attain as much statistical significance as possible by utilizing a multicenter dataset that incorporated histological information. Accordingly, the presence of glomerular crescents in patients with type 2 DKD is indicative of poor outcome. Therefore, the identification and monitoring of glomerular crescents should trigger vigilant patient management. It is imperative to undertake further large-scale prospective clinical studies and conduct in-depth investigations into the pathophysiology to enhance our understanding of ongoing and treatment-resistant DKD.

### Supplementary Information

**Supplementary Material 1.**

**Supplementary Material 2.**

## Data Availability

The datasets used and/or analyzed during the current study available from the corresponding author on reasonable request.

## Abbreviations

DKD: Diabetic kidney disease
HR: Hazard ratio
CKD: Chronic kidney disease
ESKD: End-stage kidney disease
RPS: Renal Pathology Society
eGFR: Estimated glomerular filtration rate
CI: Confidence interval
ANCA: Anti-neutrophil cytoplasmic antibody
GBM: Glomerular basement membrane
SE: Standard error
uPCR: Random urine protein-to-creatinine ratio
C3: Complement 3
C1q: Complement 1q
C4d: Complement 4d
IgA: Immunoglobulin A
IgG: Immunoglobulin G

## References

[1] Samsu N (2021). Diabetic Nephropathy: Challenges in Pathogenesis, Diagnosis, and Treatment. Biomed Res Int.

[2] Magliano DJ, Boyko EJ (2021). IDF Diabetes Atlas.

[3] Ene-Iordache B, Perico N, Bikbov B, Carminati S, Remuzzi A, Perna A (2016). Chronic kidney disease and cardiovascular risk in six regions of the world (ISN-KDDC): a cross-sectional study. Lancet Glob Health.

[4] Mise K, Hoshino J, Ueno T, Hazue R, Sumida K, Hiramatsu R (2015). Clinical and pathological predictors of estimated GFR decline in patients with type 2 diabetes and overt proteinuric diabetic nephropathy. Diabetes Metab Res Rev.

[5] Chemouny JM, Bobot M, Sannier A, Maisons V, Jourde-Chiche N, Ferriere E (2021). Kidney Biopsy in Type 2 Diabetes: A Multicenter Cross-Sectional Study. Am J Nephrol.

[6] Garcia-Martin F, Gonzalez Monte E, Hernandez Martinez E, BadaBoch T, Bustamante Jimenez NE, Praga TM (2020). When to perform renal biopsy in patients with type2 diabetes mellitus? Predictive model of non-diabetic renal disease. Nefrologia (Engl Ed).

[7] Pham TT, Sim JJ, Kujubu DA, Liu IL, Kumar VA (2007). Prevalence of nondiabetic renal disease in diabetic patients. Am J Nephrol.

[8] Tervaert TW, Mooyaart AL, Amann K, Cohen AH, Cook HT, Drachenberg CB (2010). Pathologic classification of diabetic nephropathy. J Am Soc Nephrol.

[9] Oh SW, Kim S, Na KY, Chae DW, Kim S, Jin DC (2012). Clinical implications of pathologic diagnosis and classification for diabetic nephropathy. Diabetes Res Clin Pract.

[10] Zhu X, Xiong X, Yuan S, Xiao L, Fu X, Yang Y (2016). Validation of the interstitial fibrosis and tubular atrophy on the new pathological classification in patients with diabetic nephropathy: A single-center study in China. J Diabetes Complications.

[11] Okada T, Nagao T, Matsumoto H, Nagaoka Y, Wada T, Nakao T (2012). Histological predictors for renal prognosis in diabetic nephropathy in diabetes mellitus type 2 patients with overt proteinuria. Nephrology (Carlton).

[12] Shoji T, Kanda T, Nakamura H, Hayashi T, Okada N, Nakanishi I (1996). Are glomerular lesions alternatives to microalbuminuria in predicting later progression of diabetic nephropathy?. Clin Nephrol.

[13] Zipfel PF, Wiech T, Grone HJ, Skerka C (2021). Complement catalyzing glomerular diseases. Cell Tissue Res.

[14] Tao J, Zhao J, Qi XM, Wu YG (2021). Complement-mediated M2/M1 macrophage polarization may be involved in crescent formation in lupus nephritis. Int Immunopharmacol.

[15] Trimarchi H, Barratt J, Cattran DC, Cook HT, Coppo R, Haas M (2017). Oxford Classification of IgA nephropathy 2016: an update from the IgA Nephropathy Classification Working Group. Kidney Int.

[16] Bajema IM, Wilhelmus S, Alpers CE, Bruijn JA, Colvin RB, Cook HT (2018). Revision of the International Society of Nephrology/Renal Pathology Society classification for lupus nephritis: clarification of definitions, and modified National Institutes of Health activity and chronicity indices. Kidney Int.

[17] Schroers JE, Gilbert AM, McKenzie PR, Kirwan PD, Chadban SJ, Ying T (2022). Rapidly progressive crescentic diabetic nephropathy: two case reports. Intern Med J.

[18] Otani N, Akimoto T, Yumura W, Matsubara D, Iwazu Y, Numata A (2012). Is there a link between diabetic glomerular injury and crescent formation? A case report and literature review. Diagn Pathol.

[19] Wakabayashi N, Takeda S, Imai T, Akimoto T, Nagata D (2015). Unexpected observation of glomerular crescents in a patient with diabetes who developed drug-induced acute tubulointerstitial nephritis: A possible feature of diabetic nephropathy?. Nephrology (Carlton).

[20] Morimoto M, Namba-Hamano T, Notsu S, Iwata Y, Yasuhara Y, Yamato M (2023). Diabetic nephropathy with marked extra-capillary cell proliferation: a case report. BMC Nephrol.

[21] Toth T (1987). Epithelial crescent in diabetic glomeruli. A case report Int Urol Nephrol.

[22] Sun L, Duan T, Zhao Q, Xu L, Han Y, Xi Y (2022). Crescents, an Independent Risk Factor for the Progression of Type 2 Diabetic Kidney Disease. J Clin Endocrinol Metab.

[23] Jiang S, Yu T, Zhang Z, Fang J, Wang Y, Yang Y (2019). Prognostic nomogram and score to predict renal survival of patients with biopsy-proven diabetic nephropathy. Diabetes Res Clin Pract.

[24] Saito A, Komatsuda A, Saito M, Kaga H, Abe F, Sawamura M (2020). Clinicopathological features and outcomes of diabetic kidney disease with extracapillary hypercellularity: a Japanese single-center experience. Clin Exp Nephrol.

[25] Zhao L, Liu F, Li L, Zhang J, Wang T, Zhang R (2021). Solidified glomerulosclerosis, identified using single glomerular proteomics, predicts end-stage renal disease in Chinese patients with type 2 diabetes. Sci Rep.

[26] Levey AS, Stevens LA, Schmid CH, Zhang YL, Castro AF, Feldman HI (2009). A new equation to estimate glomerular filtration rate. Ann Intern Med.

[27] Parmar MS, Bashir K (2024). Crescentic Glomerulonephritis.

[28] Naik RH, Shawar SH (2023). Rapidly Progressive Glomerulonephritis.

[29] Suarez-Alvarez B, Liapis H, Anders HJ (2016). Links between coagulation, inflammation, regeneration, and fibrosis in kidney pathology. Lab Invest.

[30] Fogo AB, Lusco MA, Najafian B, Alpers CE (2016). AJKD Atlas of Renal Pathology: Pauci-immune Necrotizing Crescentic Glomerulonephritis. Am J Kidney Dis.

[31] Chen A, Lee K, Guan T, He JC, Schlondorff D (2020). Role of CD8+ T cells in crescentic glomerulonephritis. Nephrol Dial Transplant.

[32] Anguiano L, Kain R, Anders HJ (2020). The glomerular crescent: triggers, evolution, resolution, and implications for therapy. Curr Opin Nephrol Hypertens.

[33] Flyvbjerg A (2017). The role of the complement system in diabetic nephropathy. Nat Rev Nephrol.

[34] Duan S, Sun L, Nie G, Chen J, Zhang C, Zhu H (2020). Association of Glomerular Complement C4c Deposition With the Progression of Diabetic Kidney Disease in Patients With Type 2 Diabetes. Front Immunol.

[35] Sun ZJ, Li XQ, Chang DY, Wang SX, Liu G, Chen M (2019). Complement deposition on renal histopathology of patients with diabetic nephropathy. Diabetes Metab.

[36] Elfenbein IB, Reyes JW (1975). Crescents in diabetic glomerulopathy. Incidence and clinical significance Lab Invest.

[37] Hir ML, Keller C, Eschmann V, Hahnel B, Hosser H, Kriz W (2001). Podocyte bridges between the tuft and Bowman's capsule: an early event in experimental crescentic glomerulonephritis. J Am Soc Nephrol.

[38] Gaut JP, Hoshi M, Jain S, Liapis H (2014). Claudin 1 and nephrin label cellular crescents in diabetic glomerulosclerosis. Hum Pathol.

[39] Drew AF, Tucker HL, Liu H, Witte DP, Degen JL, Tipping PG (2001). Crescentic glomerulonephritis is diminished in fibrinogen-deficient mice. Am J Physiol Renal Physiol.

[40] Stout LC, Kumar S, Whorton EB (1993). Focal mesangiolysis and the pathogenesis of the Kimmelstiel-Wilson nodule. Hum Pathol.

[41] Santoro D, Torreggiani M, Pellicano V, Cernaro V, Messina RM, Longhitano E (2021). Kidney Biopsy in Type 2 Diabetic Patients: Critical Reflections on Present Indications and Diagnostic Alternatives. Int J Mol Sci..

[42] Zoppini G, Targher G, Chonchol M, Ortalda V, Negri C, Stoico V (2012). Predictors of estimated GFR decline in patients with type 2 diabetes and preserved kidney function. Clin J Am Soc Nephrol.

[43] Kriz W, Lowen J, Grone HJ (2023). The complex pathology of diabetic nephropathy in humans. Nephrol Dial Transplant.

[44] Lowen J, Grone EF, Gross-Weissmann ML, Bestvater F, Grone HJ, Kriz W (2021). Pathomorphological sequence of nephron loss in diabetic nephropathy. Am J Physiol Renal Physiol.

[45] An Y, Xu F, Le W, Ge Y, Zhou M, Chen H (2015). Renal histologic changes and the outcome in patients with diabetic nephropathy. Nephrol Dial Transplant.

