# Supplementary material for: Glomerular crescents are associated with the risk of type 2 diabetic kidney disease progression: a retrospective cohort study
Source: BMC Nephrol. 2024 May 20;25:172. doi: 10.1186/s12882-024-03578-y (PMC11106926; doi:10.1186/s12882-024-03578-y)
Supplement: Supplementary file 1 — Supplementary Material 1. [file 12882_2024_3578_MOESM1_ESM.docx]

Supplementary Table 1. Risk factors related to kidney outcome except for glomerular crescents

| Variables | Hazard ratio (95% confidence interval) | *P* |
| --- | --- | --- |
| Age (per 1 year) | 0.96 (0.939–0.981) | <0.001 |
| Male (vs. female) | 1.02 (0.611–1.698) | 0.945 |
| Body mass index (per 1 kg/m^2^) | 1.01 (0.943–1.079) | 0.797 |
| Hypertension (vs. none) | 1.93 (1.001–3.738) | 0.050 |
| Cardiovascular disease (vs. none) | 1.38 (0.618–3.101) | 0.430 |
| Malignancy (vs. none) | 1.11 (0.493–2.484) | 0.806 |
| Hemoglobin (per 1 g/dl) | 0.93 (0.812–1.062) | 0.278 |
| Albumin (per 1 g/dl) | 0.53 (0.349–0.796) | 0.002 |
| Cholesterol (per 1 mg/dl) | 1.00 (0.997–1.004) | 0.717 |
| Blood urea nitrogen (per 1 mg/dl) | 1.00 (0.975–1.022) | 0.857 |
| Creatinine (per 1 mg/dl) | 1.72 (1.138–2.592) | 0.010 |
| uPCR (per 1 g/g) | 1.05 (0.993–1.102) | <0.001 |
| Global glomerulosclerosis |  |  |
| <20% | 1 (Reference) |  |
| 20%–49% | 1.35 (0.781–2.330) | 0.284 |
| ≥50% | 2.29 (1.140–4.589) | 0.020 |
| Mesangial expansion |  |  |
| None | 1 (Reference) |  |
| Mild | 0.69 (0.378–1.254) | 0.222 |
| Moderate | 0.61 (0.284–1.320) | 0.211 |
| Severe | 0.88 (0.443–1.736) | 0.707 |
| Interstitial fibrosis |  |  |
| None | 1 (Reference) |  |
| Mild | 1.10 (0.314–3.851) | 0.882 |
| Moderate | 1.74 (0.521–5.789) | 0.369 |
| Severe | 1.05 (0.289–3.783) | 0.946 |
| Tubular atrophy |  |  |
| None | 1 (Reference) |  |
| Mild | 2.97 (0.423–20.825) | 0.274 |
| Moderate | 5.06 (0.798–32.118) | 0.085 |
| Severe | 3.96 (0.626–25.092) | 0.144 |
| Interstitial inflammation |  |  |
| None | 1 (Reference) |  |
| Mild | 1.64 (0.774–3.487) | 0.196 |
| Moderate | 1.02 (0.461–2.262) | 0.958 |
| Severe | 0.62 (0.178–2.183) | 0.459 |
| Arteriosclerosis (vs. none) | 1.02 (0.587–1.767) | 0.947 |
| Complement 3 deposition |  |  |
| None or trace | 1 (Reference) |  |
| ≥1+ | 0.58 (0.374–0.884) | 0.012 |
| Complement 1q deposition |  |  |
| None or trace | 1 (Reference) |  |
| ≥1+ | 1.44 (0.789–2.640) | 0.234 |
| Complement 4d deposition |  |  |
| None or trace | 1 (Reference) |  |
| ≥1+ | 1.59 (0.548–4.633) | 0.392 |
| Fibrinogen deposition |  |  |
| None or trace | 1 (Reference) |  |
| ≥1+ | 1.22 (0.652–2.298) | 0.529 |
| Immunoglobulin A deposition |  |  |
| None or trace | 1 (Reference) |  |
| ≥1+ | 0.91 (0.497–1.682) | 0.773 |
| Immunoglobulin G deposition |  |  |
| None or trace | 1 (Reference) |  |
| ≥1+ | 0.80 (0.497–1.278) | 0.346 |
| Immunoglobulin M deposition |  |  |
| None or trace | 1 (Reference) |  |
| ≥1+ | 1.36 (0.887–2.084) | 0.158 |
| κ light chain deposition |  |  |
| None or trace | 1 (Reference) |  |
| ≥1+ | 0.36 (0.124–1.043) | 0.060 |
| λ light chain deposition |  |  |
| None or trace | 1 (Reference) |  |
| ≥1+ | 2.02 (0.792–5.131) | 0.141 |

uPCR, random urine protein-to-creatinine ratio.

Supplementary Table 2. Baseline characteristics of included studies

| Author (year) | Country | Case | Control | Hazard ratio (95% confidence interval) |
| --- | --- | --- | --- | --- |
| Jiang S, et al. (2019) | China | 16 | 94 | 3.55 (1.30–9.71) |
| Saito A, et al. (2020) | Japan | 22 | 48 | 0.79 (0.30–2.07) |
| Zhao L, et al. (2021) | China | 32 | 290 | 2.68 (1.55–4.62) |
| Sun L, et al. (2022) | China | 20 | 135 | 2.68 (1.27–5.64) |
