# Supplementary material for: Glomerular crescents are associated with the risk of type 2 diabetic kidney disease progression: a retrospective cohort study
Source: BMC Nephrol. 2024 May 20;25:172. doi: 10.1186/s12882-024-03578-y (PMC11106926; doi:10.1186/s12882-024-03578-y)
Supplement: Supplementary file 2 — Supplementary Material 2. [file 12882_2024_3578_MOESM2_ESM.docx]

Supplementary Table 2. Baseline characteristics of included studies

| Author (year) | Country | Case | Control | Hazard ratio (95% confidence interval) |
| --- | --- | --- | --- | --- |
| Jiang S, et al. (2019) | China | 16 | 94 | 3.55 (1.30–9.71) |
| Saito A, et al. (2020) | Japan | 22 | 48 | 0.79 (0.30–2.07) |
| Zhao L, et al. (2021) | China | 32 | 290 | 2.68 (1.55–4.62) |
| Sun L, et al. (2022) | China | 20 | 135 | 2.68 (1.27–5.64) |
